# Consumers’ experiences of back pain in rural Western Australia: access to information and services, and self-management behaviours

**DOI:** 10.1186/1472-6963-12-357

**Published:** 2012-10-11

**Authors:** Andrew M Briggs, Helen Slater, Samantha Bunzli, Joanne E Jordan, Stephanie J Davies, Anne J Smith, John L Quintner

**Affiliations:** 1 Curtin Health Innovation Research Institute, Curtin University, GPO Box U1987, PERTH, Western Australia, 6845, Australia; 2 Department of Health, Government of Western Australia, Perth, Australia; 3 School of Physiotherapy, Curtin University, Perth, Australia; 4 Pain Medicine Unit, Fremantle Hospital and Health Service, Perth, Australia

**Keywords:** Back pain, Qualitative, Education, Policy, Health services, Self-management, Rural

## Abstract

**Background:**

Coordinated, interdisciplinary services, supported by self-management underpin effective management for chronic low back pain (CLBP). However, a combination of system, provider and consumer-based barriers exist which limit the implementation of such models into practice, particularly in rural areas where unique access issues exist. In order to improve health service delivery for consumers with CLBP, policymakers and service providers require a more in depth understanding of these issues. The objective of this qualitative study was to explore barriers experienced by consumers in rural settings in Western Australia (WA) to accessing information and services and implementing effective self-management behaviours for CLBP.

**Methods:**

Fourteen consumers with a history of CLBP from three rural sites in WA participated. Maximum variation sampling was employed to ensure a range of experiences were captured. An interviewer, blinded to quantitative pain history data, conducted semi-structured telephone interviews using a standardised schedule to explore individuals’ access to information and services for CLBP, and self-management behaviours. Interviews were digitally recorded and transcribed verbatim. Inductive analysis techniques were used to derive and refine key themes.

**Results:**

Five key themes were identified that affected individuals’ experiences of managing CLBP in a rural setting, including: 1) poor access to information and services in rural settings; 2) inadequate knowledge and skills among local practitioners; 3) feelings of isolation and frustration; 4) psychological burden associated with CLBP; and 5) competing lifestyle demands hindering effective self-management for CLBP.

**Conclusions:**

Consumers in rural WA experienced difficulties in knowing where to access relevant information for CLBP and expressed frustration with the lack of service delivery options to access interdisciplinary and specialist services for CLBP. Competing lifestyle demands such as work and family commitments were cited as key barriers to adopting regular self-management practices. Consumer expectations for improved health service coordination and a workforce skilled in pain management are relevant to future service planning, particularly in the contexts of workforce capacity, community health services, and enablers to effective service delivery in primary care.

## Background

Chronic musculoskeletal pain, particularly chronic low back pain (CLBP), constitutes a significant personal and societal burden [1-3]. Although people living with painful conditions represent Australia’s third most costly health problem, less than 10% of Australians with chronic non-cancer pain, of which CLBP represents the majority [4], have access to best-practice care [5]. CLBP is the seventh most common clinical reason why people attend a general practitioner in Australia [6] and the fifth most common reason in the USA [7]. Provision of readily available evidence-based information, care and services to this group of consumers with musculoskeletal pain is important, not only to address quality of life for the consumer, but also to minimise the threat to human capital and to optimise the quality and efficiency of service delivery [5,8], especially in geographically isolated areas. Importantly, while the experience of LBP is very common [9], it is a heterogeneous condition with variable aetiology and signs and symptoms. While some presentations of LBP require targeted, interdisciplinary treatment, based on particular prognostic factors, others require much less intervention [10].

Effective, reliable, and accessible health service delivery models for consumers with musculoskeletal pain are particularly important considering the associated sequelae including poor psychosocial wellbeing [11], reduced physical activity [12] and reduced functional activity including work [13]; all of which increase both the personal and societal burden [1], particularly public health costs [14]. A growing body of evidence points to the importance of providing evidence-based information and skills along with interdisciplinary assessment and management for those consumers with CLBP, delivered in a consumer-oriented framework [13,15-17]. Further, the importance of active management, often termed ‘self-management’, in collaboration with management from health professionals, is highlighted in evidence-based clinical guidelines and consumer-based resources [15]. This approach is also reflected in Government policy, such as the Western Australian Spinal Pain Model of Care [8], a state policy for how health services should be delivered to consumers with spinal pain, and other position statements [5]. To engage in effective self-management for CLBP, consumers need to possess adequate skills, knowledge and have timely access to community resources, and the support of their healthcare providers [18-20]. In the context of CLBP, this is particularly relevant in primary care as evidence accumulates regarding a disconnect between existing practitioner treatments, behaviours and beliefs and the evidence for effective management [21-24]. Paradoxically, this gap is wider for those practitioners who profess a special interest in LBP management [25].

In Australia, individuals who live in urban areas generally have a greater range of options to access health services, while those who live in rural areas tend to be relatively disadvantaged in terms of access to multidisciplinary health services, including health services for pain [26], particularly in primary care [27]. A comparable situation has been identified in the most rural areas in Canada [28]. As a result of this asymmetric access distribution, health is generally poorer in the highly ruralised areas of Australia [29], consistent with other nations [30]. While barriers and enablers to accessing information and services related to CLBP in metropolitan Western Australia (WA) have been explored previously [19,20], similar issues have not been investigated for consumers with CLBP who reside in rural WA, where unique access issues exist.

In order to implement effective consumer-centred health services it is critical to understand what consumers identify as the barriers and enablers to accessing information and services and engaging in self-management, and to establish their needs in the context of service delivery for CLBP. Investigating these issues, particularly in areas where unique access issues exist, such as the rural sector, is important for both the individual consumer and policymakers tasked with the planning and design of healthcare systems and services and translating a well-established evidence-base and policy direction [8] for effective management into service delivery models and everyday clinical practice [31]. Qualitative methods have become more commonplace in health services research because they provide a means of exploring how people make sense of their social world and provide insights into people’s behaviour and perceptions that are not readily accessible through quantitative methods, such as surveys [32]. This allows for the development of concepts to help understand social phenomena in natural or ‘real world’ (rather than experimental) settings, giving due emphasis to the meanings, experiences and views of all participants which assists to clearly identify patient needs to inform policy and program development [33]. Therefore, the aim of this study was to qualitatively explore perceptions and behaviours of consumers with CLBP in rural WA related to access to information and services for CLBP and their self-management behaviours.

## Methods

### Participants

Fourteen participants (5 male, 9 female; mean (SD) age: 57.0 (13.8) years, range 35–77 years) were involved in this qualitative study. These 14 participants represented a subset (27%) of 52 consumers who attended group-based pain self-management education forums held throughout rural WA, in 2010 and 2011; they also participated in a prospective evaluation of the forums [34]. All consumers self-selected to attend the forums by responding to community-based advertisements (newspapers, local radio advertisements, referral from local health professionals) and enrolled through the event organiser, Arthritis and Osteoporosis WA ( http://www.arthritiswa.org.au). In this context, rural refers to towns greater than 200 km from Perth, which is the capital city of WA. The forums were a condensed version of the Self-Educative Pain Sessions (STEPS) programme, developed and evaluated previously in WA [35] and aimed to provide evidence-based information and skills to consumers regarding self-management and co-care for spinal pain conditions, consistent with recommendations from the WA Spinal Pain Model of Care [8].

The participants in this study were recruited from forums held in Kununurra (n=4) (2210 km from Perth, population: 5600), Albany (n=5) (409 km from Perth, population: 23900) and Kalgoorlie (n=5) (538 km from Perth, population: 36800), and consented to participate in a semi-structured telephone interview.

### Sampling

While there were no criteria imposed for enrolling to attend one of the forums, inclusion criteria for this study required that consumers experienced CLBP (pain ≥ 3 months duration) and had adequate skills in written and spoken English. No minimum levels of pain or disability criteria were imposed for this study. Human Research Ethics Committees at the Western Australian Country Health Service and Curtin University approved the study and the study complied with the Declaration of Helsinki. All participants provided written, informed consent to participate and were assured of their anonymity in any publication and confidentiality and security of their responses. Maximum variation sampling was used to recruit participants for this study. The purpose of this method is to sample a range of participants who have diverse characteristics based on criteria selected *a priori*[36]. Among those forum attendees who consented to participate in a telephone interview, participants for this study were selected based on their average CLBP intensity in the last week (reported in demographic and clinical questionnaires at the commencement of the forums) and satisfaction rating with the forum (reported at the completion of the forum). Average CLBP intensity was measured using an 11 point numeric rating scale (NRS) from 0 to 10, with the left anchor 0 = no pain and the right anchor at 10 = maximal imaginable pain [37]. Satisfaction was measured using a Global Perceived Impression of Usefulness Scale, scored on an 11 point NRS from 0 to 10, with the left anchor 0 = not at all useful and the right anchor at 10 = extremely useful [38]. As part of the maximum variation sampling, the gender distribution was considered to ensure the proportion of females in this qualitative study (64.3%) was consistent with the broader prospective study (68.8%) [34]. All consumers who were invited to participate in this qualitative study consented to do so.

### Data collection and interviews

At the forums, attendees completed questionnaires about their first language, country of birth, education level, employment status, access to Government benefits, insurance claims for their condition, consultations with health professionals, and average intensity of their CLBP. These data were used to characterise the cohort and enable maximum variation sampling for this qualitative study.

Interviews were conducted 12 weeks after participants attended a forum. The interview schedule was developed in consultation with expert musculoskeletal and pain management clinicians, policy makers, researchers and consumers of the WA Musculoskeletal Health Network ( http://www.healthnetworks.health.wa.gov.au/network/musculoskeletal.cfm). The Network is a multidisciplinary group of people (carers, consumers, clinicians, educators, researchers) with a shared interest in improving service delivery in Western Australia for musculoskeletal health. The schedule was developed to explore with consumers three key areas, including: access to information about CLBP; access to health services for CLBP; and current self-management behaviours for managing CLBP. Questions were developed around each of these key areas with input from the research team and also following consultation through members of the WA Musculoskeletal Health Network. Questions were designed to provide meaningful information regarding spinal pain management which could be used by policy makers and health service designers in the planning of health service delivery for rural areas in WA. A trained interviewer, independent of the forum presentations and who remained blinded to the participants’ demographic, socioeconomic, and pain history characteristics conducted the telephone interviews. All interviews were recorded and transcribed verbatim. The interviewer (SB) was trained by an experienced qualitative researcher (JEJ). Some standardised prompts were used throughout the interviews, when required, to stimulate responses with respect to self-management strategies and access to information. For example, probes for questions related to accessing services included examples like access to swimming pools, gymnasiums, and local health centres.

### Data analysis

Data analysis was undertaken in stages, commencing with full verbatim transcriptions of interviews and then content analysis of interview transcripts by one author (SB), using inductive techniques to thematically code and develop categories according to trends identified from the transcripts [39]. All interviews were then reviewed independently by two other authors (AMB, JEJ). Syntheses from the three researchers were compared and categories debated where necessary to reach consensus. This process included looking at how categories linked together, identifying clear differences between categories and careful examination of the data to ensure that categories had been saturated. This led to the development and refinement of key themes. The three authors involved in reviewing the transcripts agreed that saturation of themes was achieved with the study sample.

## Results

**Figure 1:**
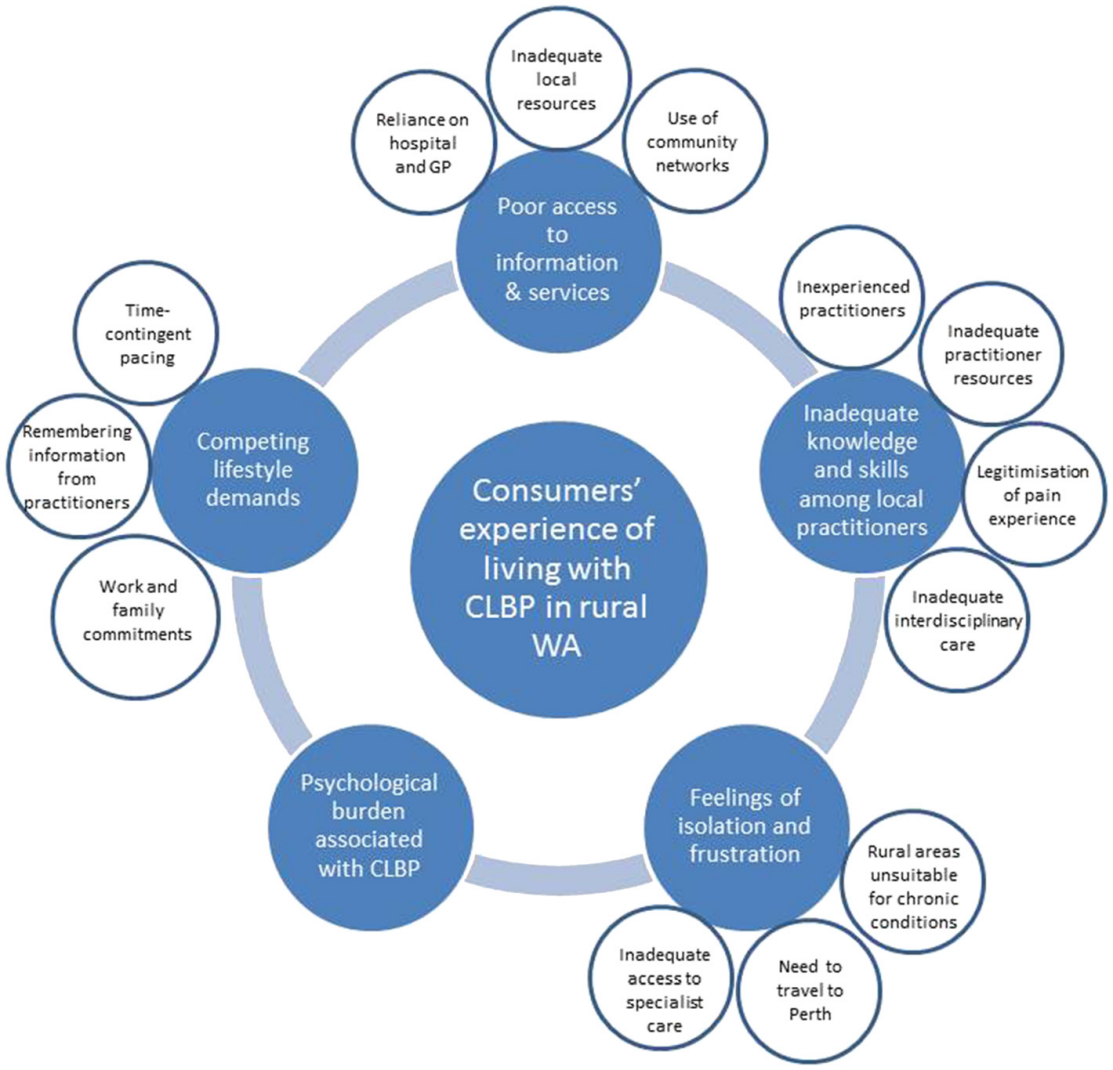
**Schematic showing the five major themes influencing consumers’ experiences of living with CLBP in rural WA.** The factors which contribute to these major themes are also displayed.

Fourteen interviews were conducted, ranging from 8–32 minutes in duration. Table 1 provides a summary of the demographic and clinical data collected from the participants when they registered to attend a forum. The five key themes are described below and summarised in Figure 1. There was strong consistency between researchers in the identified themes. Quotes which specifically aligned to the identified themes have been included.

**Table 1:** Demographic and clinical characteristics of the participants

**Characteristic**	
English as a first language, n (%)	13 (92.9)
Born in Australia, n (%)	7 (50.0)
Highest education level achieved, n (%)	
Year 10 or less	2 (14.3)
TAFE / Vocational college	1 (7.1)
Year 12	4 (28.6)
University qualification	7 (50.0)
Currently employed, n (%)	6 (42.9)
Access to an Australian Healthcare card^, n (%)	7 (50.0)
Current insurance claim for injury, n (%)	1 (7.1)
Have consulted a health professional for LBP, n (%)	14 (100.0)
Average LBP intensity measured by NRS‡, mean (SD), min-max.	
Current	2.9 (2.8), 0-8
In the last week	5.2 (2.6), 1-9
In the last month	5.1 (2.1), 2-8
Satisfaction with forum measured by GPIU† NRS, mean (SD), min-max	6.5 (3.0), 0-10

^ The Health Care Card assists Government benefit recipients, low income earners and selected other groups with access to cheaper Government-subsidized prescription medicines and a lower Extended Medicare Safety Net threshold.

‡ NRS: numeric rating scale.

†GPIU: global perceived impression of usefulness.

### Poor access to information and services in rural settings

A major theme to emerge from participants was the lack of adequate, consumer-centred community-based resources (both information and services) for the management of pain-related conditions, especially LBP. For the majority of participants, this conclusion had been reached through numerous attempts to locate community-based resources in their local area, although some individuals indicated that they did not know where to initiate searching for information and services.

"“… [services or information available] nothing, nothing at all! I don’t even know where to look, you know, I mean I ask around and nobody seems to know.” (Female participant (KA1), Kalgoorlie)"

"“Well the information is just not there; it’s not available. Or, I suppose it is, if you know where to look, but there is nobody saying anything, giving you a pamphlet, and saying this is where you go for support services, so I don’t even know what is available in Albany.” (Female participant (AL1), Albany)"

Some participants cited local community networks of friends, family and colleagues as a useful mechanism to source information and services for LBP, usually based on ‘word of mouth’.

"“… he [allied health practitioner] lives at the end of our street and I walk past him all the time and you know how you get referred to people by friends, well he has a really good name by lots of sports people that we know.” (Female participant (KA5), Kalgoorlie)"

However, despite access to community networks participants encountered a similar experience of not being able to identify any relevant information from their local networks.

"“I have asked people my own age group what is what, and where to get a doctor, and all that sort of thing, and what’s up there, what do you get up at the hospital, and that sort of thing, and people, the ones I have asked, they don’t know.” (Female participant (KA1), Kalgoorlie)"

Consequently, individuals relied heavily on their local hospital or general practitioner (GP) to be the primary provider of services for their LBP. Nonetheless, participants indicated that neither the hospital nor GP were equipped with the necessary resources to assist with non-acute healthcare needs related to CLBP.

"“Well I’m in a pretty remote area where there are no services at all basically. There has been a GP service here for the last 2 years, before that it would only be the Kununurra hospital and they were pretty much emergency treatment only.” (Male participant (KU2), Kununurra)"

"“I still find dealing with the GP quite difficult, quibbling if you want pain relief and things like that. I certainly have got some medication, but I feel like it’s very reluctantly given.” (Female participant (AL5), Albany)"

### Inadequate knowledge and skills among local practitioners

Another related factor that affected participants’ ability to access relevant information and services was the perception that local healthcare providers lacked adequate knowledge and skills in comprehensive management of pain disorders, particularly chronic pain disorders. Participants attributed this situation to inexperienced practitioners working in rural areas, such as new graduates, and/or health professionals not having the adequate resources, such as time, professional networks, and knowledge of contemporary evidence, to assist individuals with a chronic condition.

"“We often get first year grads and I know first year grads are excited and full of information but they often don’t have the experience.” (Female participant (KA5), Kalgoorlie)"

"“Look, I love my GP, he is really good, but he is not a pain specialist. He is not, not skilled in pain, but he is just not up to date in everything to do with pain, because pain is now really becoming a discipline in its own right.” (Male participant (KA2), Kalgoorlie)"

"“It’s not derogatory on them, it’s just that they don’t know back pain as a specialty, it’s just that they are always under pressure, they are always under-staffed and under pressure and it is always the new recruits who come out to the country areas and none of them have specialist experience in back pain.” (Male participant (KA2), Kalgoorlie)"

Participants also expressed frustration about not having their pain experience recognised as real and important by health professionals. Based on their experience, individuals often discussed that access to effective care for their CLBP from their health professional (usually a GP) was largely influenced by two factors. The first was finding a practitioner who legitimised their condition.

"“It all depends on your doctor, which is very frustrating. You go to a certain GP and they will put you on a Patient Assisted Transport form and they will fly you because of pain, and not wanting to sit on a train for seven hours, but it depends on your GP, which it shouldn’t.” (Female participant (KA5), Kalgoorlie)"

"“… it took me a long time, probably 18 months, which sounds dreadful, to find a GP that would listen and knew that you were real, because you know, pain is very difficult to pinpoint.” (Female participant (KA5), Kalgoorlie)"

"It’s not like having an arm in a sling, or something, so people can be prepared. If they can see it, or if you walk with a bit of a limp or something like that, or crutches, people are a bit more sympathetic.” (Female participant (AL3), Albany)"

The second factor cited by participants as influencing the adequacy of care was having a health provider who would provide holistic care and was prepared to coordinate and integrate care with other non-medical practitioners such that a cross-discipline management plan was developed. Participants expressed large levels of frustration at the poor communication between health providers and that well-coordinated interdisciplinary care was not commonplace.

"“Something I have always found quite difficult is getting reliable information or guidance about managing pain in a kind of holistic sense. I can go to a physio, I can go to a chiropractor, I can go to a doctor, I can take medication, and obviously I can search on the internet, but trying to work out how things come together has always been a bit of a problem.” (Female participant (AL2), Albany)"

"“I just found that the doctors were fairly inclined to say, you know that there are people worse off than you, you’ll be alright or alternatively prescribe stronger and stronger pain killers, which is not what I want. I wasn’t saying that I need pain killers, I was saying that I need to know how to start getting a management plan together, that sort of response is not helpful at all for me.” (Female participant (AL2), Albany)"

"“What I would have liked from my GP in the first instance was perhaps a suggestion then to talk to an OT [occupational therapist], to use various parts of your body to not put undue stress on your joints; maybe a suggestion, I don’t know, whether there is an arthritis nurse or something like that locally. Just something, so I knew the GP was hearing my concerns and was helping me with a management plan.” (Female participant (AL2), Albany)"

### Feelings of isolation and frustration

The majority of participants were resigned to the belief that nothing could be done for their condition because they lived in isolated, rural areas. Many perceived that rural settings were better suited to younger people who would be less likely to need access to healthcare services, particularly for chronic conditions associated with pain.

"“These sorts of remote areas, they are for fit young people you know, not for silly old military veterans like me. It’s not a disabled veteran’s sort of country. It’s purely an area for young people”. (Male participant (KU2), Kununurra)"

"“There is a lady here who has to fly down to Perth every two weeks or something, it’s just not an area where that sort of thing [health infrastructure] is realistic you know”. (Male participant (KU2), Kununurra)"

Despite acceptance of their isolation, participants were still highly frustrated that they needed to travel to Perth to access adequate pain-related health services which incurred cost, time and inconvenience.

"“It’s frustrating because for like a pump refill I need to fly down in the morning [to Perth], I have to go to xxxx street, the pharmacy that makes up the script, then go to Dr X’s rooms, then wait until lunchtime and then get him to do the pump refill and then go back to the airport and sit around for 4 or 5 hours until the plane is ready to go. So, it’s frustrating, it takes a whole day to do a 15 minute thing!” (Male participant (KA2), Kalgoorlie)"

"“. it [travelling to Perth] still means time off for my husband who has got to take leave [from work]. For the last few years the only holiday we have taken together is all to go to Perth together. 700km in the car is not that much fun with little ones.”(Female participant (KA5), Kalgoorlie)."

"“…you have to pay your own accommodation and cos’ I can’t drive that distance I have to pay for my flights down and my own accommodation and all that sort of thing.” (Male participant (KA2), Kalgoorlie)"

Participants identified a clear need for improved access to specialists, particularly pain specialists, and a local workforce skilled in pain management. The use of telemedicine was identified as a possible solution to overcome local workforce limitations and a mode of consultation that suited pain medicine.

"“Specialist treatment is pretty much non-existent. You have to wait two or three months for a visiting specialist or get yourself off to Darwin or Perth or somewhere, or some major centre” (Male participant (KU2), Kununurra)"

"“I call him up [pain specialist] and the secretary says you have to make an appointment and I say I just want to talk to him, I don’t need to see him. That is tricky with pain, because you can talk on the telephone, because you don’t need to be examined, so that [telemedicine] would be nice.” (Female participant (KA5), Kalgoorlie)"

### Competing lifestyle demands hindering effective self-management for CLBP

Competing lifestyle demands, such as work and family commitments, were cited as substantial barriers to participants adopting a regular self-management routine utilising self-perceived or practitioner-prescribed coping strategies to manage their LBP, such as periodic rest, exercise (swimming, stretching, therapeutic exercises), and medication.

"“… it’s just trying to access [the pool]; I have a little one so it’s just getting out and finding the time to do these things.” (Female participant (KA5), Kalgoorlie)"

"“I like doing them [stretches and exercises], and I know I have to, but sometimes it is just like, you come home from work and you’ve got to feed the dogs, and make some food and do the dishes and then its bed time.” (Female participant (KU3), Kununurra)"

Participants identified that the concept of pacing activity in a time-contingent manner was a coping strategy very difficult to integrate into their lifestyles.

"“… some people, they have to just get these things done, and they don’t have the option to [pace].” (Female participant (KU3), Kununurra)"

"“….not pushing through your pain which is the most USELESS advice you can give to anybody because you have to push through the pain just to survive unless you live in a nursing home where somebody does everything for you.” (Female participant (AL1), Albany)"

### Psychological burden associated with CLBP

A few participants referred to the strain their CLBP experience placed on multiple aspects of their life and wellbeing, including comorbidity, which led to feelings of hopelessness and compounding the belief that ‘nothing can be done’.

"“… the fact that it is not just pain, it affects you in every single way because it is constant pain so it affects you psychologically too because you waste so much time going to doctors and specialists and on medication…” (Female participant (KA5), Kalgoorlie)"

"“… it [the body] all seems to be, you know, falling apart at this time in my life. It is frustrating because when I came here [rural site] I really wanted to get out into the community and do something. I thought even last night, what can I do? It has to be something sitting down, you know, it’s frustrating.” (Female participant (KA1), Kalgoorlie)"

## Discussion

### Main findings

This qualitative study revealed barriers to information and services encountered by consumers with CLBP in rural WA, and captured their self-management behaviours. Difficulties in accessing a local, skilled workforce and the limited availability of community-based resources were particularly pronounced, although many of the issues identified mirror those experienced by consumers in urban, metropolitan WA [20]. Consumer expectations for improved access to health services for pain, including access to specialists, coordination of interdisciplinary health services and improved workforce capacity in the area of pain management are relevant to future service planning, particularly in the contexts of workforce capacity, community services, and enablers to effective service integration such as eHealth and primary care reform initiatives. The psychosocial impact of living with CLBP in a rural setting was emphasised by participants and represents an important issue for consideration in service design and clinical interactions. Importantly, consumer expectations align with recommendations in the Australian National Pain Strategy [5].

### Access to LBP information and services

Consistent with international data [40], GPs were nominated by consumers as the primary source for information about CLBP. This contrasts to information-seeking behaviours among consumers in urban WA, who viewed physiotherapists and chiropractors as the health professionals who could provide more comprehensive information about the management of CLBP [20]. Considering allied health workforce limitations in rural Australian areas [41,42], it is not surprising that GPs were identified as the primary source of information for this cohort. Nonetheless, GP-initiated management strategies were reported to be more focused on providing short-term symptomatic relief, usually through prescription medications, rather than addressing self-management support, spreading care coordination across multidisciplinary providers and working with consumers and other health professionals to create mutually agreeable management plans. This situation exists despite there being evidence for the effectiveness of a multidisciplinary mode of service delivery for consumers with CLBP [40,43], particularly in rural settings [44], and that almost one fifth of GP consultations relate to chronic pain [45]. A substantial barrier encountered by GPs to providing a more holistic service, however, is likely to be a lack of time. For example, the average consultation length for an Australian GP is in the order of 12–13 minutes in a rural practice [46]. Nonetheless, consultation time is generally longer for GPs in rural practices compared to metropolitan practices and when dealing with chronic health problems, compared to non-chronic health problems [47]. The need for a more holistic service from GPs in rural areas is consistent with data from another recent study which examined the perceived needs of consumers with chronic pain in a primary care setting [48]. This need is also supported by evidence that the cost-effectiveness for LBP management could be significantly improved when multidisciplinary services were added to usual GP care [40] and additional cost efficiencies are achievable when guideline-consistent interventions for LBP are implemented, compared with costs of alternative interventions [49]. A lack of service co-ordination was cited by consumers as a significant barrier to accessing effective management for their CLBP, suggesting that it is critical for health professionals to have access to, and knowledge of, interdisciplinary referral pathways. These barriers may be overcome, in part, by encouraging health professionals (and health students) to practice and train within an interdisciplinary framework, for example, within a community of practice or network model [50]. Ameliorating this lack of access and any associated knowledge gaps may help to address the barrier cited by respondents concerning the lack of experience and knowledge among rural health professionals for managing consumers with CLBP. Furthermore, better systems to optimise care coordination, rather than just self-management, for consumers should remain a priority for future health planning, particularly in the context of reform primary healthcare.

Reasons underlying the disconnect between evidence and practice in this rural context are likely to be multifactorial [42]; including, current workforce limitations, lack of community-based services, lack of systems to facilitate care coordination, lack of professional development opportunities, lack of awareness of clinical guidelines [51,52] and evidence [15], lack of medical practitioners working in a team-care model [53], and appropriate funding models; all issues identified in relation to pain services and documented in the National Pain Strategy [5]. While it is recognised that a paradigm shift in the management of CLBP is needed [54] multifaceted initiatives targeting reform of the health system; specifically, developing workforce capacity (volume and skills) and up skilling consumers are critical approaches required to facilitate improved management of CLBP [5,8,15].

Increasing consumers’ and health professionals’ knowledge and skills about management strategies for CLBP is a potential avenue to drive positive behavioural health change for consumers with CLBP [54,55]. Findings from targeted educational programmes for consumers [56] and health professionals [57] using similar evidence-based information appropriately titrated for each cohort, support the effectiveness of this interprofessional approach. Importantly, these approaches should recognise the psychosocial sequelae and comorbidities associated with persistent pain and better equip practitioners to recognise, assess and manage psychosocial contributions to persistent pain. Recent data from France also indicate that GP behaviours can be modified for the management of LBP through the delivery of a simple information campaign [58]. Although providing information and skills is important, the capacity to translate knowledge into real-world practice is critical and relies on skills in system navigation – an important component of health literacy [20]. System navigation relates to the capacity of health system users – both health professionals and consumers - to locate and use appropriate health information and services at the right time. Respondents in our study cited that barriers to undertaking active management were the paucity of local information resources. Health literacy, the capacity to seek, understand and use health information [59] is essential for effective management of chronic health conditions [60], including CLBP [20]. Our findings highlight the need to provide consumers with readily accessible, sustainable, contemporary and practical, usable, evidence-based information. Web-based resources may provide one opportunity to enable delivery of information to suit a variety of consumer learning styles [61], and may be an efficient and sustainable mode of service delivery for both consumers and health professionals, particularly in rural settings. Such resources are likely to also benefit clinicians.

Consumers cited a significant barrier to accessing pain-services was the lack of legitimisation of their pain experience by health professionals and their community. The National Pain Strategy and Declaration of Montréal both emphasise the right for people in pain to access appropriate health services, and that pain should be recognised as a chronic condition in its own right [5,62]. Therefore, a societal legitimisation of the experience of pain is important and might be facilitated through broad public health initiatives, policies, and targeted education for health professionals, particularly those working in primary care. Such approaches might limit the significant and negative consequences associated with stigmatisation [63], and ultimately help to improve the care for consumers living the experience of pain.

### Self-management behaviours

Despite evidence for the benefits of consumers using active management strategies to manage their pain and to optimise their outcomes [64,65], in our study competing social, financial and lifestyle factors were identified by consumers with CLBP as key barriers to effective integration of self-management strategies into daily life. These factors mirror reports about self-management from other consumers with persistent pain [19,66-68]. Consistent identification of these factors highlights the critical importance for clinicians to help consumers implement tailored solutions to help enable positive health behaviour change [18,19]. At a system level, these data reinforce the importance of self-management support for consumers [69], with the aim of enabling a greater uptake of community-based active self-management approaches [65]. While we did not collect detailed data on the self-management strategies adopted by consumers in this qualitative study, we have reported quantitative data about specific self-management strategies for LBP adopted by consumers in rural WA [34].

Individual beliefs drive pain behaviour [20,67,70] and unhelpful beliefs have a significant and negative association with consumer outcomes [71]. Therefore, targeted strategies aimed at optimising consumers’ helpful beliefs related to the effective management of CLBP are critical in order to improve acceptance and implementation of active management strategies into their lifestyle. At a population level, mass media campaigns may be useful [72], while at the individual level, providing simple, evidence-based, consumer-centred information while discussing access options and priorities with individual patients is important [68].

A strong theme to emerge in our study was the difficulty consumers experienced in applying the principles of pacing into their everyday lives. While pacing to avoid the ‘boom-bust’ cycle is advocated clinically [73], implementation remains challenging for consumers, reportedly because of competing work and lifestyle demands. In our study, data suggest that pacing may not be readily accepted unless it can be more therapeutically integrated into existing lifestyles [68]. Further, to improve function through graded activity exposure unless pacing is adopted in a time-contingent, rather than pain-contingent manner, the effects may be provocative, reinforce unhelpful behaviours including activity avoidance, and contribute to greater disability [74]. These context-driven and definition-specific aspects of pacing activity may account for the inconsistency in evidence related to the efficacy of pacing [75]. Factors may also include the priority allocated to the task over competing activities of daily living.

### Strengths and limitations

The strength of this study lies in its qualitative approach. Recent studies have highlighted the advantages of qualitative methods for capturing detailed information in health research [20,76]. This format allows patients to speak in their own voice and identify the barriers to accessing information and services and implementing effective self-management behaviours for CLBP without conforming to categories and terms imposed on them by others. These data relate specifically to the availability of health information and services in WA, and therefore should be interpreted in that context. Further, we acknowledge that participants in this study self-selected to attend the education forums and this may present an element of responder bias, since more motivated consumers may have attended. Consequently, we are unable to speculate on the self-management behaviours of consumers in rural sites who are potentially less motivated to attend educational forums. While the sites where data were collected differ in geography and population size, similar themes emerged by site during the analysis. However, the themes identified in this research are by no means all encompassing. As with all qualitative research, there is only an ability to represent reality rather than present the ultimate truth [77]. It may be important in future studies to purposively sample based on sociodemographic characteristics and geography in order to enable comparisons between different rural settings. Further, it may be important to understand in more detail the specific barriers experienced by rural consumers in adopting positive behaviour change to manage CLBP.

## Conclusion

Consumers in rural WA experienced barriers in accessing information and services, particularly specialist and interdisciplinary services, for CLBP and implementing self-management practices into everyday life. Consumer expectations for improved health service coordination and improved workforce capacity are relevant to future service planning, particularly in the contexts of workforce capacity, community health services, and enablers to effective service delivery in primary care.

## Abbreviations

CLBP: Chronic low back pain; GP: General practitioner; NRS: Numeric rating scale; OT: Occupational therapist; WA: Western Australia.

## Competing interests

The authors declare they have no competing interests.

## Authors’ contributions

AMB, HS, SJD, JLQ conceived the study and developed the design and methods. JEJ contributed to the development of qualitative methods. AMB, HS, SJD and JLQ procured funding. AMB, HS, SB, SJD, AJS, JLQ were responsible for data collection. AMB, HS, SB, JEJ, AJS were responsible for data analysis and reporting of the results. All authors contributed to the preparation of the manuscript. All authors read and approved the final manuscript.

## Pre-publication history

The pre-publication history for this paper can be accessed here:

http://www.biomedcentral.com/1472-6963/12/357/prepub
